# Genome assembly of a Mesoamerican derived variety of lima bean: a foundational cultivar in the Mid-Atlantic USA

**DOI:** 10.1093/g3journal/jkab207

**Published:** 2021-07-23

**Authors:** Randall J Wisser, Sara J Oppenheim, Emmalea G Ernest, Terence T Mhora, Michael D Dumas, Nancy F Gregory, Thomas A Evans, Nicole M Donofrio

**Affiliations:** 1 Department of Plant and Soil Sciences, University of Delaware, Newark, DE 19716, USA; 2 Laboratoire d’Ecophysiologie des Plantes sous Stress Environnementaux, INRAE, Univ. Montpellier, SupAgro, 34060 Montpellier, France; 3 Sackler Institute for Comparative Genomics, American Museum of Natural History, New York, NY 10024, USA; 4 Cooperative Extension, University of Delaware, Georgetown, DE 19947, USA

**Keywords:** Lima bean, common bean, resistance genes, partial resistance

## Abstract

Lima bean, *Phaseolus lunatus*, is closely related to common bean and is high in fiber and protein, with a low glycemic index. Lima bean is widely grown in the state of Delaware, where late summer and early fall weather are conducive to pod production. The same weather conditions also promote diseases such as pod rot and downy mildew, the latter of which has caused previous epidemics. A better understanding of the genes underlying resistance to this and other pathogens is needed to keep this industry thriving in the region. Our current study sought to sequence, assemble, and annotate a commercially available cultivar called Bridgeton, which could then serve as a reference genome, a basis of comparison to other *Phaseolus* taxa, and a resource for the identification of potential resistance genes. Combined efforts of sequencing, linkage, and comparative analysis resulted in a 623 Mb annotated assembly for lima bean, as well as a better understanding of an evolutionarily dynamic resistance locus in legumes.

## Introduction

Lima bean (*Phaseolus lunatus* L.) was independently domesticated in Mesoamerica and the Andes (Motta-Aldana *et al.* 2010; Serrano-Serrano *et al.* 2012) and is now cultivated throughout the world. Lima bean is high in fiber, protein, and slow-release carbohydrates, making it a healthy, low glycemic index food (Bello-Pérez *et al*. 2007).

In the United States, lima bean is grown predominantly in the Mid-Atlantic, specifically New Jersey, Delaware, and Maryland. In Delaware alone, lima bean production is approximately a $9.8 million industry (USDA-NASS, 2017). The same conditions that are conducive to robust pod production, however, are also conducive to the development and proliferation of several oomycete pathogens, including pod rot caused by *Phytophthora* *capsici* (Davidson *et al.* 2002, 2008; Evans *et al.* 2007) and downy mildew caused by *Phytophthora phaseoli*. In 1998, a new race of *P. phaseoli* emerged that decimated lima bean production in Delaware, prompting breeding for resistance genes and increased studies on this important plant, and its pathogens (Evans *et al.* 2002, 2007).

In 2016, Mhora *et al*. used bulked segregant analysis (BSA) to map a resistance locus effective against the predominant field race F of *P. phaseoli*. To our knowledge, no other resistance loci have been mapped in lima bean. Collinearity analysis with the *P. vulgaris* (common bean) genome revealed homology with a resistance gene (*R*-gene) dense region containing different subtypes of nucleotide-binding site leucine-rich repeat (NLR) genes (Schmutz *et al.* 2014). Without a reference genome for *P*. *lunatus*, Mhora *et al*. were unable to compare the gene content in lima bean. Thus, the limited genomic resources for lima bean have hindered further dissection of this region of the genome and genome-wide analysis of disease resistance.

In addition to *R*-gene mediated defense, research into lima bean diversity and the genetic basis of more complex traits will require comprehensive genomic data resources. Therefore, we sequenced the *P. lunatus* cultivar Bridgeton, a variety of Mesoamerican origin that was favored by farmers in the Mid-Atlantic USA until the emergence of *P. phaseoli* race F. Furthermore, we generated linkage and syntenic maps to form larger scaffolds, and we performed QTL analysis of a slow mildewing phenotype that provides partial resistance against *P. phaseoli* (Santamaria *et al.* 2018). As a new reference genome of lima bean, this report provides a foundation for future work.

## Materials and methods

### Biological materials

A single seed of Bridgeton, namely “Bridgeton-DES4” (reproduced from NPGS PI 549508), was chosen to self-propagate seed for the reference genome. Progeny of Bridgeton-DES4 was grown in continuous darkness to generate etiolated tissue for DNA extraction. Separate extractions were performed on tissue from individual plants using the Qiagen Maxi DNA extraction kit, followed by a DNA purification (Greco *et al.* 2014). The DNA was checked for purity using the NanoDrop ND-1000 spectrophotometer (Thermo Fisher Scientific, MA, USA) and quantified using both Picogreen (Thermo Fisher Scientific, MA, USA) and QUBIT (Thermo Fisher Scientific, MA, USA). Fragment analysis and agarose gel electrophoresis were used to confirm the presence of nonfragmented, high molecular weight DNA.

### Sequencing

The DNA from a single, high-quality Bridgeton-DES4 plant extract was shipped to NRGene (San Diego, CA, USA) and subjected to library construction according to their protocols. Five size fractions were selected ranging from 470 bp to 10 kb to construct sequencing libraries following the manufacturer’s protocols (Illumina, San Diego, CA, USA). The TruSeq DNA Sample Preparation Kit version 2 with no PCR amplification (PCR-free) was used to make replicate paired-end libraries for the 470 and 800 bp size fractions. The Nextera MP Sample Preparation Kit was used to make mate-pair (MP) libraries with 2–5, 5–7, and 7–10 kb jumps. The 470-bp libraries were sequenced as 2 × 265 nucleotides on the Hiseq2500 v2 in rapid mode. The 800-bp libraries and part of the three MP libraries were sequenced as 2 × 160 bp nucleotides on the HiSeq2500 (v4 Illumina chemistry) while the remainder of the MP libraries were also sequenced as 2 × 150 bp on the HiSeq4000. A total of 246 Gb sequencing data (equivalent to ∼360X genomic coverage, based on an estimated genome size of 686 Mb). All library construction and sequencing were performed at Roy J. Carver Biotechnology Center, University of Illinois at Urbana-Champaign.

### Assembly

Genome assembly was conducted using the DeNovoMAGIC^™^ software platform (NRGene, Ness Ziona, Israel). This is a De Bruijn graph-based assembler, designed to efficiently extract the underlying information in the raw reads to solve the complexity of the DeBruijn graph due to genome polyploidy, heterozygosity, and repetitiveness. This task is accomplished using accurate-reads-based traveling in the graph that iteratively connects consecutive phased contigs over local repeats to generate long phased scaffolds (Lu *et al.* 2015; Hirsch *et al.* 2016; Avni *et al.* 2017; Zimin *et al.* 2017; Zhao *et al.* 2017).

In brief, the algorithm included the following steps:

1. Preprocessing: PCR duplicates, Illumina adaptor AGATCGGAAGAGC, and Nextera linkers (for MP libraries) were removed. The PE 450 bp 2 × 265 bp libraries overlapping reads were merged with minimal required overlap of 10 bp to create the stitched reads.
2. Error correction: Following preprocessing, merged PE reads were scanned to detect and filter reads with putative sequencing error (contain a subsequence that does not reappear several times in other reads).
3. Contigs assembly: The first step of the assembly consists of building a De Bruijn graph (kmer = 127 bp) of contigs from all of the PE and MP reads. Next, PE reads were used to find reliable paths in the graph between contigs to resolve repeats and extend the contigs.
4. Scaffolds assembly: Contigs were linked into scaffolds with PE and MP information, estimating gaps between the contigs according to the expected distance of PE and MP links.
5. Fill Gaps: A final gap-filling step used PE and MP links and De Bruijn graph information to detect a unique path connecting the gap edges.

### Linkage and syntenic maps

Linkage and synteny mapping approaches were used to cluster and order the assembled scaffolds into draft pseudomolecules. Linkage mapping was performed using genotyping-by-sequencing (GBS) data on 163 F_2_ progeny from a cross between cultivars Cypress and Jackson Wonder. 192-plex sequencing libraries were constructed following the protocol by Manching *et al.* (2017) using RASP-2.0 adapters redesigned for *Csp*6I/*Msp*I and *Csp*6I/*Taq*^α^I pairs of restriction enzymes. Samples included the F_2_ progeny along with replicate samples of the parents and F_1_ plants as well as a negative control with no DNA. Separate libraries were constructed for *Csp*6I/*Msp*I and *Csp*6I/*Taq*^α^I. Sequencing was performed at the University of Delaware’s Sequencing and Genotyping Center on a Hiseq2500 run in rapid mode at 1 × 151 bp.

Processing of GBS data was performed using RedRep (https://github.com/UD-CBCB/RedRep). First, FASTQ files from two lanes of sequencing were merged for the corresponding libraries. Following quality control and barcode deconvolution, FASTQ files from the same barcode were merged and then mapped to the NRGene sequence assembly. Variants were typed using HaplotypeCaller, and the genotype matrix was filtered as follows. VCFtools (Danecek *et al.* 2011) was used to set genotype calls to missing if fewer than three reads supported the call. The resulting genotype matrix was processed in R version 3.4.1 (R Core Team 2018) with custom scripts to filter markers that (1) had greater than 75% missing data; (2) had inconsistent genotype calls between parental replicates; (3) were heterozygous in either parent; (4) did not have the expected parent-hybrid trio genotypes; and (5) had F_2_ allele frequencies less than 15% or greater than 85%.

Using sequencing scaffolds containing at least five markers, missing data were imputed using LB-Impute (Fragoso *et al.* 2016). A linkage map was constructed with QTL IciMapping software v 4.1.0.0 (Meng *et al.* 2015) [run settings: DIS (20 cM) grouping function; RECORD ordering algorithm; SARF (window size = 5) rippling criterion].

Chromosomer (Tamazian *et al.* 2016) was used to align the NRgene scaffolds against the *P*. *vulgaris* reference genome (Schmutz *et al.* 2014) downloaded from JGI (“*Pvulgaris_442_v2.0.softmasked.fa.gz*”). First, while retaining softmasked sequences in the reference genome identified by analysis with RepeatMasker, LAST (Kiełbasa *et al.* 2011) was used to identify and softmask additional repeat sequences using the NEAR seeding scheme (run settings: -uNEAR and -R11). Following guidelines for human-ape alignments (https://github.com/mcfrith/last-genome-alignments, last accessed December 2019), substitution and gap frequencies were determined with last-train (run settings: –revsym –matsym –gapsym -E0.05 -C2) and lastal alignment was performed (run settings: -fMAF -K2 -m50 -E0.05 -C2, and -p corresponded to the output from last-train). Alignments between repeat sequences were discarded with last-postmask and a python script was used to retain only the top two LAST matches per query sequence. The resulting output was used to run fragmentmap and assembler routines of Chromosomer. Finally, LAST was used to align the Chromosomer assembled *P*. *lunatus* genome to the *P*. *vulgaris* reference genome (run settings: -m50 -E0.05 -C2).

### QTL analysis

Disease reaction to race F of downy mildew (caused by *P. phaseoli*) was determined for 384 F_2:3_ families of the Cypress (resistant; slow mildewing phenotype) X Jackson Wonder (susceptible) cross (163 of the F_2_ parents of these families were used to construct the linkage map; see above). Plants were grown in a greenhouse humidity chamber and inoculated at emergence as described by Santamaria *et al.* (2018). All families were replicated across sequential plantings (four batches across time) with five plants per family in a single pot in each round. Pots were arranged in a randomized incomplete block design with six subblocks augmented with repeated checks of resistant, tolerant, and susceptible varieties. Individual plants were measured for lesion length on the stem and rated for the quantity of sporulation on a 1–5 scale. To account for differences in stem length, lesion length was standardized by plant height. Adjusted means for sporulation rating and height-standardized lesion length on the F_2:3_ progeny was used as an estimate of the corresponding F_2_ parent phenotype for QTL analysis. QTL IciMapping software v 4.1.0.0 (Meng *et al.* 2015) was used to perform inclusive composite interval mapping with an LOD threshold of 2.5 and step size of 1 cM.

### Gene annotation

Prior to structural annotation, repetitive elements were identified and masked (Supplementary File S1) using RepeatMasker (Smit *et al.* 2013) with a custom library that included all *Viridiplantae* entries from Repbase (Bao *et al.* 2015) along with repetitive elements from *P. vulgaris* (Gao *et al.* 2014). Using the masked assembly, gene predictions were generated by AUGUSTUS with the *Arabidopsis thaliana* training set (Keller *et al.* 2011; Scalzitti *et al.* 2020). The completeness of the genome assembly and AUGUSTUS gene models was analyzed with BUSCO, which measures completeness in terms of evolutionarily informed expectations of gene content (Simão *et al.* 2015). The BUSCO Embryophyta dataset (1440 single-copy conserved genes) was used as a reference.

Annotation of the predicted genes was undertaken with multiple tools. First, BLASTp searches (-evalue 1e-10) were performed against a custom BLAST database that included all Viridiplantae sequences from the NCBI nr database (Wheeler *et al.* 2008). For sequences that had no hit, a second BLASTp search was performed against all sequences in the nr database. There were 14 RNA-Seq libraries in PRJNA596114 (*P*. *lunatus* genome), generated from pods (12 libraries), leaves (1 library), and flowers (1 library). We used MagicBlast at NCBI to map these reads to the predicted genes. The resulting SAM file was converted to BAM format and indexed, followed by samtools idxstats to identify predicted genes with either no transcript coverage or those that were covered over their full length. For the remaining genes with partial coverage, bedtools genomecov was used to calculate the fraction of transcript coverage across each gene. In addition, using BLASTp (-evalue 1e-10), the predicted proteins for *P*. *lunatus* were compared to the *P*. *vulgaris* reference proteome (https://www.uniprot.org/proteomes/UP000000226, last accessed July 2020).

We used protein domain analysis to annotate NLR genes. To support comparison with common bean, we followed the same procedure for annotating NLRs described by Schmutz *et al.* (2014).

## Results and discussion

**Figure 1:**
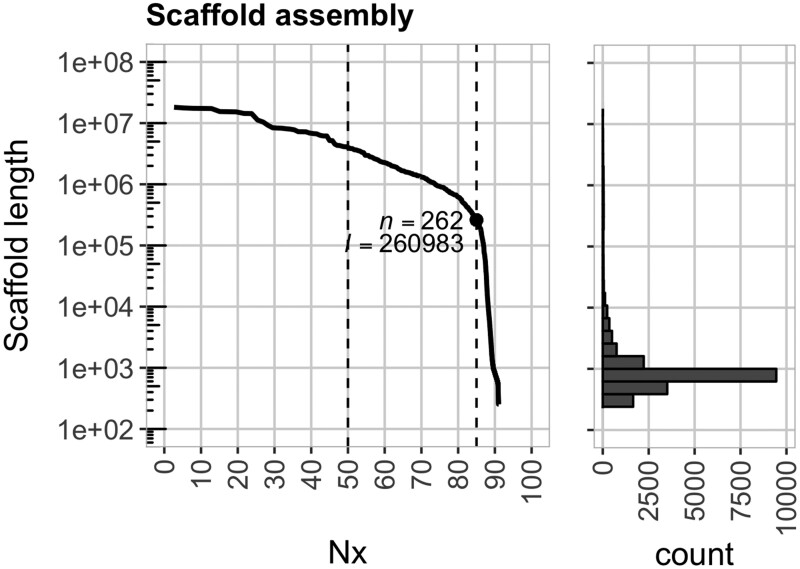
Summary statistics for the assembly of lima bean cultivar Bridgeton. The plot on the left shows the scaffold length as a function of the contiguity value (Nx). The N50 and N85 are marked by the dashed vertical lines where the corresponding number (*n*) and cumulative length (*l*) of scaffolds are noted. The marginal histogram plot on the right shows the number of scaffolds at different lengths.

We report a 623 Mb genome assembly of the Mesoamerican lima bean cultivar Bridgeton. This constitutes ∼91% of the 686 Mb genome estimated by flow cytometry for *P. lunatus* (Pellicer and Leitch 2020). The final assembly has an average depth of ∼70X (Supplementary Table S1) and is comprised of 19,316 scaffolds, 36 and 262 of which captured 50% and 85% of the expected genome size, respectively (Figure 1), with a corresponding N50 of 3.99 Mb and N85 of 0.26 M. The N50 was 5.17 Mb with respect to the sequence space (as opposed to the expected genome size). Consistent with the nucleotide composition of higher plant genomes, the GC content for lima bean was 38%, the same as its closest relative with a reference genome, common bean (Schmutz *et al.* 2014). The genome was analyzed for completeness, returning a BUSCO score of 93% (Table 1). Although this represents a nearly complete genome assembly for lima bean, the assembly is comprised of thousands of scaffolds that vastly exceed the 11 (2*n* = 22) chromosomes of *P*. *lunatus* (Bonifácio *et al.* 2012).

**Table 1:** Genome statistics and annotated gene data

Total scaffolds	19,316
Total contigs	58,167
Complete BUSCOs	1,341 (93%)
Scaffolds with predicted proteins	3,417
Protein-coding genes	64,541
Genes with transcript evidence	40,308
Proteins with P. vulgaris hit	32,505
Proteins with other Viridiplantae hit	1,356
Proteins with non-Viridiplantae hit	2,363
Proteins with no hit in nr database	28,317

Linkage and synteny mapping methods were used to further tether the assembly scaffolds. Using GBS data, a linkage map was constructed from 942 markers present in 46 scaffolds that captured ∼50% of the sequence space. The largest 11 linkage groups constituted a 670 cM map (see MAP directory in Supplementary File S2) comprised of 898 markers among 41 scaffolds (25 of which were in the 36 N50 set and all of which were within the N85 set) that together captured 49% of the sequence space. In every case, markers within a given scaffold mapped to a single linkage group. However, within LGs 1, 3, and 5, markers were ordered on the genetic map such that sections of different scaffolds interleaved with other scaffolds (Supplementary Table S2).

Synteny analysis with the common bean genome (Schmutz *et al.* 2014) ordered 3839 of the *P. lunatus* scaffolds, which were distributed among all 11 *P*. *vulgaris* chromosomes (Supplementary File S3). These corresponded to many different scaffolds in the *P*. *lunatus* assembly (∼230 Mb or 37% of the assembled genome) than those anchored to the genetic map. There were 16 scaffolds anchored by both maps which comprised ∼105 Mb. Together, nearly 70% of the sequence space was ordered by genetic mapping or synteny analysis, but with only 25% intersection, these separate maps could not be well integrated. Additional work will be required to construct chromosome-scale pseudomolecules. Nevertheless, as described below, combining all of the map data helped to identify a homologous section of the *P. lunatus* and *P. vulgaris* genomes associated with variation in disease resistance.

We mapped a QTL on LG 9 (*PlPp_LG9.1*) that explained ∼50% of the phenotypic variation in partial resistance to *P*. *phaseoli* (see BIP directory in Supplementary File S2), a slow downy-mildewing phenotype. Previously, with a different population, Mhora *et al.* (2016) used BSA to map a race-specific major effect, race-specific gene also associated with resistance to *P*. *phaseoli*. Anchoring the marker sequences from QTL and BSA mapping onto our genome assembly showed that both of these resistance loci reside at the same region of the genome, indicating they are linked or that *PlPp_LG9.1* is a weak allele of the race-specific resistance gene.

Markers present in scaffold 25,456 were associated by both QTL and BSA mapping. Two additional scaffolds contained BSA-associated markers, but these scaffolds were absent from the linkage map used for QTL analysis [due to marker QC filtering (scaffold 3610) and failure to join a linkage group (scaffold 974); scaffold 974 contained markers with the strongest BSA association]. Synteny mapping helped to delimit the physical section of the genome containing both resistance loci, but this required a lowering the standard sensitivity threshold for chromosomer (-r 1.01; default is 1.2). Based on these results, 15 scaffolds spanned the QTL and BSA-associated loci with flanking markers in scaffolds 3610 and 25,456 (see Supplementary File S4 fragment map). Consistent with previous findings (Mhora *et al.* 2016), this corresponded to a homologous section on chromosome 4 in *P*. *vulgaris* (coordinates: 75,772–1,552,787). The approximate length of *P*. *lunatus* scaffold sequences between the flanking markers was 1.3 Mb, smaller than *P*. *vulgaris* by ∼175 kb.

### Genome-wide annotation

Augustus predicted 64,541 genes from the masked assembly (Supplementary File S5). As is typical of draft genome assemblies, this is likely an overestimation of the true gene number in *P. lunatus*. Inflated gene counts can result from assembly fragmentation (a single gene sequence spread across multiple contigs) and failure to join distant exons together in a single transcript (Denton *et al.* 2014). The BUSCO analysis also suggests overestimation of the true gene number: ∼13% of the core genes were either duplicated or fragmented.

**Figure 2:**
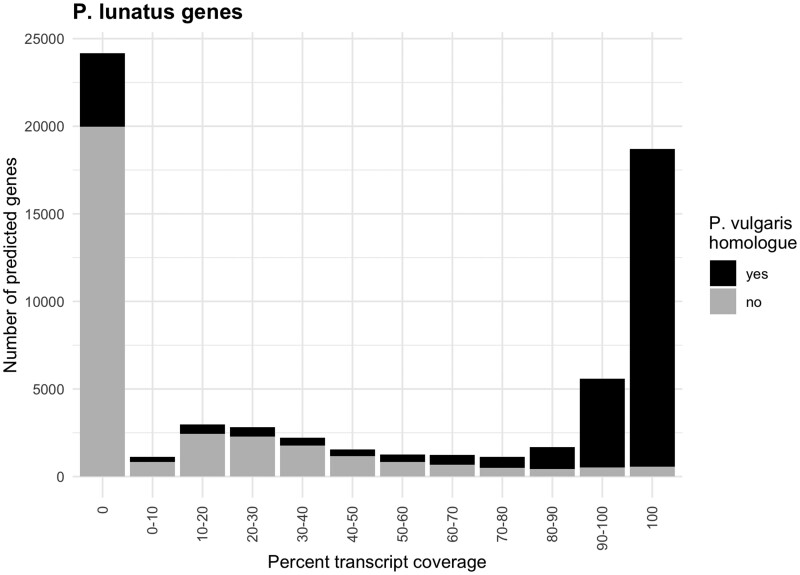
Transcript and comparative assessment of predicted genes in the Bridgeton genome. A histogram of the percent transcript coverage for predicted genes in the Bridgeton assembly. The fraction of encoded protein homologs in the common bean is indicated per class.

We compared the predicted proteins for *P. lunatus* to protein annotations for *P. vulgaris*, which diverged from *P. lunatus* ∼4 mya (Delgado-Salinas *et al.* 2006; Bitocchi *et al.* 2017). The *P. vulgaris* genome has an estimated size of 587 Mb, with 98% of the sequence anchored to 11 pseudomolecules that contain 27,197 protein-coding genes supported by transcripts (Schmutz *et al.* 2014). We found that ∼50% (32,505) of the *P. lunatus* proteins had hits to *P. vulgaris*, which encompassed 96% of the *P. vulgaris* proteins (Supplementary File S6). Using public transcriptome data for lima bean, we found that the vast majority of *P*. *vulgaris* homologs had transcript evidence in *P*. *lunatus* (Figure 2; Supplementary File S7), while most, but not all, of the nonhomologous genes were likely to be errors in *de novo* gene prediction. Of the 32,036 predicted *P. lunatus* proteins without a match in *P. vulgaris*, 28,317 proteins had no hits to any sequence in the nr database and tended to be shorter (mean of 237 aa) than those with hits (mean of 514 aa). However, 1356 had hits to other Viridiplantae species, and another subset of 2363 had hits to non-Viridiplantae entries in the nr database that were predominantly bacterial sequences. There were 903 hits to Enterobacteriaceae, which are the dominant members of many phyllosphere bacterial communities (Cernava *et al.* 2019), 359 hits to Rhizobiaceae, a family of plant-associated bacteria (Spaink *et al.* 2012), and 254 hits to Flavobacteria, which are thought to contribute to plant growth and protection (Kolton *et al.* 2016). Only 132 of the non-Viridiplantae nr hits were to eukaryotes (90 metazoa, 31 fungi, 6 Alveolata, 2 Stramenopiles, 2 Euglenozoa, and 1 Pyrenomonadales).

### Resistance genes

One of our objectives in sequencing the lima bean genome was to catalog R-genes commonly associated with disease resistance in plants. The majority of these are NLR genes defined by two major domains: a centrally located nucleotide-binding site domain, which has ATPase activity (NB-ARC) and a C-terminus leucine-rich repeat domain (LRR) (van Ooijen *et al.* 2008; Takken and Goverse 2012), but additional non-NLR types with coiled-coil domains (CNs and CNLs) are also important. The *P. vulgaris* genome contained 376 such *R*-genes (Schmutz *et al.* 2014), while the *P. lunatus* Bridgeton assembly contained 190, with the major difference due to a paucity of NLRs with a Toll/Interleukin Receptor-1 domain in lima bean (Supplementary File S8). The *P. vulgaris* genome has three particularly large clusters containing some 40 R-genes at the ends of chromosomes 4, 10, and 11 (Richard *et al.* 2018). The chromosome 4 cluster, referred to as the *B4* locus, is homologous with the locus described in this study that was associated with partial and complete resistance to downy mildew. The *B4* locus in common bean has been noted for ectopic recombination resulting in the accumulation of CNLs on chromosome 4. The homologous section in lima bean contained many fewer *R*-genes (9 compared to 39 in the common bean), but six of these, all of which were located on scaffold 974, contained a coiled-coil domain (Supplementary File S8).

## Conclusions

Closing a major gap in resources for lima bean, this study reports the reference genome for a *P*. *lunatus* cultivar that was foundational to lima bean production in the east coast. *De novo* protein predictions showing high similarity to 96% of the encoded genes for *P*. *vulgaris* (common bean) corresponded to 32,505 genes in lima bean, most of which were supported by transcriptome data (Figure 2). The sequenced variety, Bridgeton, was the primary founder of modern cultivars for the Mid-Atlantic. In this region of the USA, diseases limit the production of lima bean. Using the genome assembly, we consolidated genetic map data for loci associated with partial, race nonspecific resistance and complete, race-F-specific resistance to *P. phaseoli*, the causal agent of downy mildew. These loci colocalized in a segment that aligns to a section of chromosome 4 of common bean which is enriched with canonical *R*-genes and genetic associations with resistance to different diseases—the *B4* *R*-gene cluster (David *et al.*^2008^, ^2009^). Despite many fewer *R*-genes at the homologous locus in the Bridgeton genome, the presence of coiled-coil type *R*-genes is a shared feature. This is consistent with prior findings of ectopic recombination events at the *B4* locus that predates the divergence of lima and common bean (David *et al.* 2009). Thus, the reference genome of lima bean enabled the identification of a putative hotspot for the evolution of resistance alleles, which merits further research. This report provides a new genomic resource for investigations into the diversity and evolution of legumes.

## Data Availability

The genome sequence data for this studyis available under the NCBI BioProject PRJNA647124, for BioSample SAMN15394833 (*P*. *lunatus* cv. Bridgeton). Raw read data from the individual libraries at the Sequence Read Archive include: SRX9040258, SRX9040257, SRX9040256, SRX9040255, SRX9040254, SRX9040253, SRX9040252, SRX9040251. Supplementary material is available at figshare: https://doi.org/10.25387/g3.14398910. This Supplementary material includes eight files: a repeat masked version of the genome assembly (Supplementary File S1); the linkage map and QTL mapping results from IciMapping (Supplementary File S2); synteny maps from Chromosomer (Supplementary Files S3 and S4); all genes predicted by Augustus (Supplementary File S5); proteins of *P*. *lunatus* with a match to a *P. vulgaris* protein (Supplementary File S6); transcript coverage for *P*. *lunatus* predicted genes (Supplementary File S7); and *P.lunatus* *R*-gene annotations and comparison with *P. vulgaris* (Supplementary File S8).
